# The Effect of Puncture Sites of Portal Vein in TIPS with ePTFE-Covered Stents on Postoperative Long-Term Clinical Efficacy

**DOI:** 10.1155/2019/2935498

**Published:** 2019-01-09

**Authors:** Si-liang Chen, Peng Hu, Zhi-peng Lin, Jian-bo Zhao

**Affiliations:** ^1^Department of Interventional Radiology, Guangdong Second Provincial General Hospital, Guangzhou, 510317 Guangdong, China; ^2^Department of Interventional Radiology, Nanfang Hospital, Southern Medical University, Guangzhou, 510515 Guangdong, China; ^3^Department of Interventional Radiology, The First Affiliated Hospital of Guangxi University of Chinese Medicine, Nanning, 530023 Guangxi, China

## Abstract

**Purpose:**

To evaluate the effect of puncture sites of the portal vein in transjugular intrahepatic portosystemic shunt (TIPS) on long-term clinical efficacy.

**Methods:**

A retrospective review was performed, including consecutive 171 patients who underwent TIPS with ePTFE-covered stents. All patients were divided into 3 groups according to the puncture site of the portal vein: intrahepatic bifurcation of the portal vein (group A, *n* = 88), right branch of the portal vein (group B, *n* = 48), and left branch of the portal vein (group C, *n* = 35). The Kaplan-Meier analysis was performed to assess the effect of different puncture sites on primary patency, the incidence of hepatic encephalopathy (HE), and survival.

**Results:**

The primary restenosis rate was 29.8% (51/171). The total HE rate was 31.6% (54/171). The cumulative death rate was 19.3% (33/171). The Kaplan-Meier analysis showed that group C versus group A, group C versus group B, and group A versus group B were significantly different on the primary restenosis rate, respectively (*χ*^2^ = 11.49, *P* = 0.001; *χ*^2^ = 4.54, *P* = 0.033; and *χ*^2^ = 4.12, *P* = 0.046), and group C is better than the other two groups. What is more, group C versus group A and group C versus group B were significantly different on the incidence of HE, respectively (*χ*^2^ = 8.07, *P* = 0.004; *χ*^2^ = 9.44, *P* = 0.002), and group C is better than the other two groups. There was no significant difference on survival.

**Conclusion:**

Choosing the left branch of the portal vein as the puncture site to create the shunt in TIPS with ePTFE-covered stents may decrease the incident of primary restenosis and HE significantly.

## 1. Introduction

Transjugular intrahepatic portosystemic shunts (TIPS) has evolved into an effective and durable nonsurgical option in the treatment of portal vein hypertension [1–4]. The communication of the portal and systemic venous circulations is achieved through interventional radiology techniques after a percutaneous transjugular approach. The artificial parenchymal channel is fixed open by means of an expandable metallic stent [4]. However, there are three major potential risks in this procedure: hepatic encephalopathy (HE), shunt dysfunction, and liver failure.

The portal vein puncture is one of the most crucial procedures in TIPS [5]. Traditionally, TIPS insertion means the creation of a shunt through the hepatic parenchyma connecting one of the branches of the portal vein, most frequently the right one, with a branch of the hepatic vein, usually the right or the middle one [6]. There may be following reasons which cause different puncture sites of the portal vein. From anatomical reason and technical difficulty, most TIPS are performed by selecting the right branch or intrahepatic bifurcation of the portal vein, as they are easy to be punctured from the hepatic vein. From operator preference, some tended to agree that there was no necessity to choose the puncture site deliberately if the compliance of shunts was satisfactory, while some believed that patients with the optimal stent position could acquire better long-term clinical efficacy. However, there have been no unified clinical criteria on the puncture site of the portal vein so far, and relevant reports are limited. With the improvement of operating techniques, the long-term postoperative clinical efficacy should be taken into more consideration. Our study aims at evaluating the effect of puncture sites of the portal vein in TIPS on long-term clinical efficacy.

## 2. Materials and Methods

### 2.1. Patients

**Figure 1:**
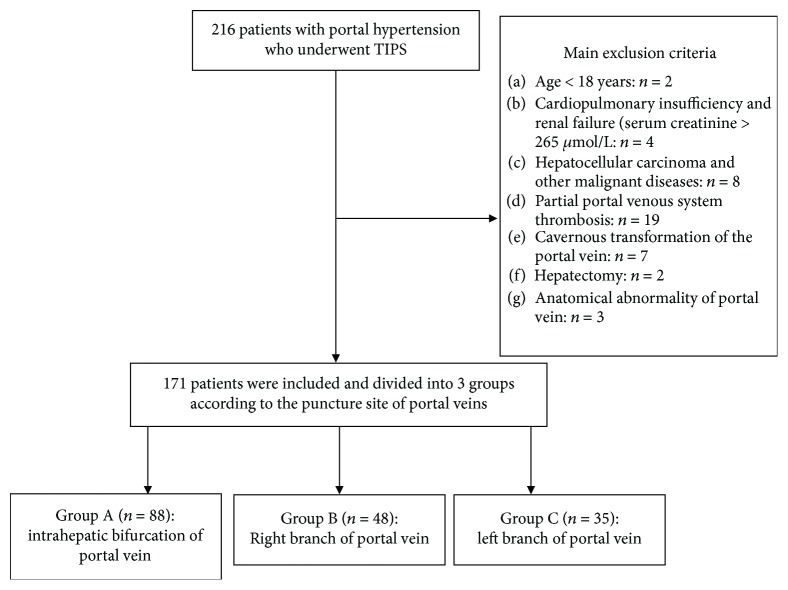
Consolidated Standards of Reporting Trials diagram of the allocation of patients to study groups.

A retrospective analysis of clinical data of all consecutive patients with symptomatic portal hypertension, repeated variceal bleeding, or refractory ascites, who established TIPS shunts using expanded polytetrafluoroethylene- (ePTFE-) covered stents from January 2010 to December 2015, was performed in the present study. A total of 171 patients were available for evaluation. The criteria are shown in Figure 1.

### 2.2. Preoperative Management

All clinical data were obtained by means of medical history and clinical laboratory tests. The liver function data in each patient were evaluated by using the Child-Pugh score and the model for end-stage liver disease (MELD) score. Abdominal three-dimensional angiograms reconstructed by means of contrast medium-enhanced computed tomography were obtained to define the anatomic relationship between the portal and hepatic veins, evaluate vascular patency, and rule out hepatocellular carcinoma. The patients with gastroesophageal varices were treated by acid suppression and hemostasis and received fluid infusion and blood transfusion to correct or prevent hemorrhagic shock. The patients with refractory ascites received human serum albumin supplementation and diuresis, and large volume paracentesis was performed if necessary. All enrolled patients received antibiotic prophylaxis periprocedurally; 0.75 g cefuroxime was infused intravenously 30 minutes before the procedure and three times daily after the procedure for 3 days [7].

### 2.3. TIPS Procedure

The TIPS creation technique has been described previously [8–10]. After indirect portography (mesenteric artery angiography) was performed, the catheterization of the right or middle hepatic vein was performed through the right internal jugular vein with a transjugular liver access set (RUPS-100; Cook Inc.). The puncture needle was advanced through the liver parenchyma from the hepatic vein to one of the branches of the portal vein, and then a guidewire was passed into the portal vein guiding percutaneous transhepatic puncture was used to assist in passes when necessary. After direct portography was performed and the portosystemic gradient (PSG) was measured, the parenchymal tract between the hepatic vein and portal vein was dilated by a 6 mm × 80 mm angioplasty balloon catheter (Powerflex; Cordis Inc.). Firstly, a 6 or 8 mm bare stent (E-Luminexx; Bard Inc.) was implanted according to the distance between the hepatic vein and the portal vein. The length of the bare stent inside the portal vein was 2 cm. Then a 6 or 8 mm ePTFE-covered stent (Fluency; Bard Inc.) was implanted. The length of the covered stent inside the portal vein was less than 1 cm. The covered stent combined with a bare stent extended to the junction of the hepatic vein and inferior vena cava. The stent shunt was dilated by a 6 or 8 × 60 mm balloon catheter (Powerflex; Cordis Inc.). In patients with a varicose gastric coronary vein, coil (Cook Inc.) embolization was performed in varices that continued to fill after TIPS creation. Finally, post-PSG measurement and portal vein angiography were performed.

### 2.4. Judgment of Puncture Site

**Figure 2:**
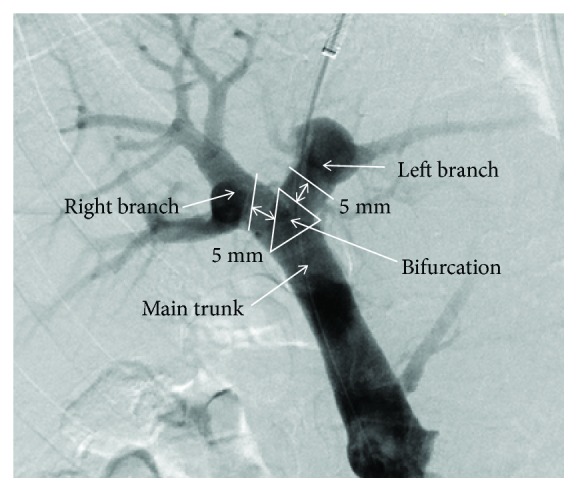
TIPS inserted within 5 mm from the portal vein bifurcation were considered bifurcation TIPS, while those inserted 5 mm greater from the bifurcation were considered branches of the portal vein.

**Figure 3:**
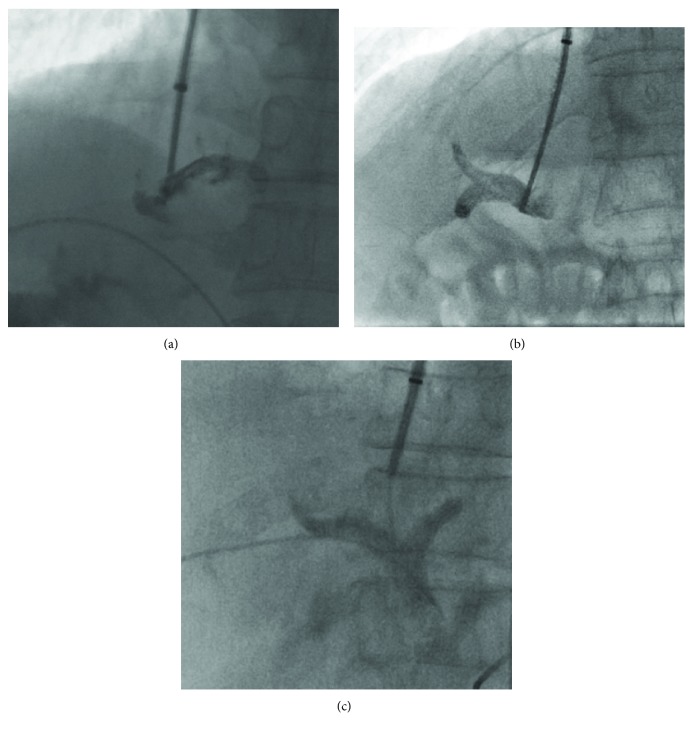
(a) If both branches or the trunk of the portal vein developed to the same extent, the intrahepatic bifurcation would be punctured. (b) If the right branch of the portal vein developed first, the right branch would be punctured. (c) If the left branch of the portal vein developed first, the left branch would be punctured.

TIPS inserted within 5 mm from the portal vein bifurcation were considered bifurcation TIPS, while those inserted 5 mm greater from the bifurcation were considered branches of the portal vein (Figure 2). For an experienced operator, the puncture sites could be appropriately selected and successfully punctured, which had been proven in previous studies [11, 12]. The puncture sites were determined by interventional radiologists and recorded before and during the TIPS procedure. The selected puncture site should be ensured that the shunt would be straighter and both ends of the stent would not be excessive bending, and operator should try to avoid the formation of stent-graft longitudinal undeformation and angulation or block both ends of the stent. Before puncturing the portal vein, the anatomical position between the hepatic vein and portal vein was estimated by CT imaging and indirect portography. The portal vein was punctured under the guidance of digital subtraction angiography in both the posterior anterior and the lateral positions. After the success of portal vein puncture, the contrast medium was injected through a paracentetic needle. The puncture site was located at the branch of the portal vein which developed first. If both branches or the trunk of the portal vein developed to the same extent, the intrahepatic bifurcation would be punctured. If the right branch of the portal vein developed first, the right branch would be punctured. If the left branch of the portal vein developed first, the left branch would be punctured (Figure 3). Moreover, the puncture sites could be confirmed further according to the balloon impressions in liver parenchymal and direct portal venography. The abdominal three-dimensional computed tomography scan would be performed to further confirm the puncture sites after TIPS if necessary.

### 2.5. Postoperative Management

After successful TIPS implantation, all patients received the medical monitoring with clinical, biochemical, and color Doppler ultrasound evaluation. The treatment for improving liver function was regularly performed. Lactulose (10 mL, three times per day) was regularly given orally to all patients for 7 days in order to prevent HE, and no further use was approved unless patients were diagnosed with HE. 0.75 g cefuroxime was infused intravenously three times daily after the procedure for 3 days. Anticoagulation was not routinely recommended except in patients treated for thrombosis of the hepatic veins. Antiplatelet therapy (Plavix, 75 mg, once a day, a total of 6 months) was carried out if platelet count is more than 80 × 10^9^/L.

### 2.6. Follow-Up

All patients were followed up in the outpatient clinic with clinical, biochemical, and color Doppler ultrasound evaluation, initially at 1 month after TIPS, then at 3 months, and every 6 months thereafter, to observe the function of the liver and kidney as well as the shunt patency of patients. Combined with the telephone follow-up, the middle-term and long-term survivals as well as complications were observed. Patients were followed from the date of diagnosis until last clinical evaluation, liver transplantation, or death.

### 2.7. Statistical Methods

Statistical analysis was performed using SPSS 20.0 statistical software (SPSS, Chicago, IL). Data are presented as the mean ± standard deviation ($\bar{\chi }\pm s$) for quantitative variables and as absolute numbers for qualitative variables. Categorical variables were compared using Fisher's exact test or the *χ*^2^ test, and continuous variables were compared with a *t*-test or one-way ANOVA. The post-TIPS primary shunt patency rate, incidence of HE, and survival rate were calculated using the life table method, assessed using Kaplan-Meier curves and compared using the log-rank test. All variables were included in univariate analyses. A Cox proportional regression hazards model was used to assess the prognostic value of the significant variables found in the univariate analyses. *P* value < 0.05 was considered statistically significant.

## 3. Results

### 3.1. Patient Characteristics

Baseline data were comparable among the three groups (*P* > 0.05) (Table 1). All patients included 125 males and 46 females, ranging 25~79 years, with the mean being 51.9 ± 12.2 years. They were followed for 6~104 months with the mean of 47.23 ± 19.71 months and the median follow-up time being 34 months. The preoperative symptoms were esophageal gastric variceal bleeding (138 patients) or refractory ascites (patients ineffective to high-dose diuretic therapy, 33 patients), of whom 139 patients were with posthepatitic cirrhosis of hepatitis B and C, 19 patients were with alcoholic cirrhosis, and 13 patients were with unknown etiology. The preoperative Child-Pugh score of liver function was 5~13 points, with the mean being 7.54 ± 1.72 points. All patients underwent abdominal computed tomography scan before TIPS, to identify the anatomical relationship among the portal vein, hepatic vein, and inferior vena cava.

### 3.2. Perioperative Period

The intrahepatic shunts were established successfully in all patients. A total of 371 stents were implanted, including 182 ePTFE-covered stents and 189 bare stents. The preoperative PSG was 24.87 ± 3.65 mmHg, while postoperative PSG was 11.57 ± 3.15 mmHg, with a significant difference between them (*t* = 19.33, *P* < 0.01). During the perioperative period, 2 patients died of acute hepatic failure and 2 patients died of acute gastrointestinal tract hemorrhage in a week after TIPS. HE was observed in 7 patients in a week after TIPS, whose symptoms were all relieved after medical treatment. Intraperitoneal hemorrhage, a fatal complication secondary to extrahepatic portal vein puncture during TIPS creation, occurred in 2 patients, including one relieved with medical treatment and one performed exploratory laparotomy for hemostasis. One case of refractory ascites was unresponsive to TIPS until the supplement of albumin and the use of diuretics.

### 3.3. Postoperative Shunt Patency

**Figure 4:**
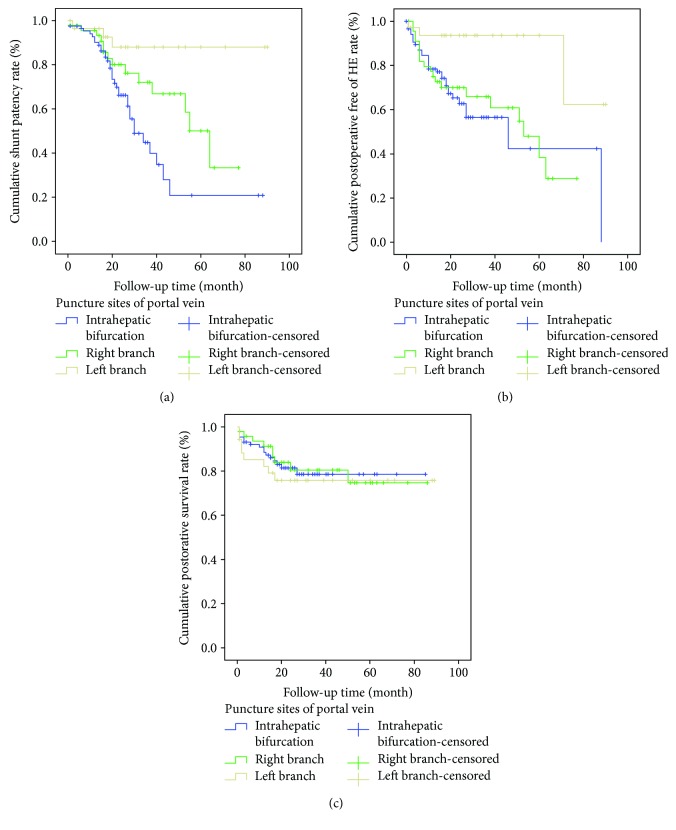
Kaplan-Meier curves for shunt patency (a), postoperative HE (b), and postoperative survival (c) in the study. The rates of cumulative shunt patency and postoperative HE were significantly different among the three groups (*P* < 0.05), while the cumulative postoperative survival did not show any difference (*P* > 0.05).

During the follow-up period, the total in-stent stenosis rate was 29.8% (51/171; group A: 38.6%; group B: 29.2%; and group C: 8.6%) (Table 2). The life table analysis showed that the 1~5-year patency rates of shunts were 89%, 81%, 73%, 55%, and 46%, respectively. The cumulative rates of shunt patency among the three groups were significantly different (*χ*^2^ = 13.40, *P* = 0.001). Kaplan-Meier analysis indicated that group C versus group A, group C versus group B, and group A versus group B were significantly different, respectively, in shunt patency (*χ*^2^ = 11.49, *P* = 0.001; *χ*^2^ = 4.54, *P* = 0.033; and *χ*^2^ = 4.12, *P* = 0.046) (Figure 4(a)). It revealed that the shunt patency of the three groups was ranked as follows: group C > group B > group A. In 51 patients, the in-stent stenosis occurred in 2~65 months after TIPS creation (21.18 ± 18.46 months), presenting as the relapse of preoperative symptoms, including haematemesis (15 cases), melena (23 cases), refractory ascites (4 cases), and no blood flow signal in the stent from color Doppler ultrasound (9 cases). Among those with in-stent stenosis, 35 underwent TIPS recanalization and 3 underwent parallel TIPS (two in group A and one in group B) (Table 3); 5 were relieved by medical treatment; 8 patients died of variceal rebleeding because the condition of rebleeding deteriorated so quickly that medical treatment was ineffective. Of 35 patients in whom TIPS recanalization was done, 2 developed stenosis again, in which 1 of them underwent a second TIPS recanalization whereas the other one refused TIPS operation.

After univariate analysis (Table 4) and multivariate analysis, the stent diameter (*P* = 0.007) and puncture site of the portal vein (*P* = 0.015) were identified as independent predictors of primary shunt patency (Table 5).

### 3.4. Postoperative HE

The total incidence of HE was 31.6% (54/171; group A: 35.2%; group B: 39.6%; and group C: 11.4%) (Table 2). The life table analysis showed that the 1~5-year HE rates were 24%, 34%, 36%, 45%, and 56%, respectively. The cumulative rates of HE among the three groups were significantly different (*χ*^2^ = 10.20, *P* = 0.006). Kaplan-Meier analysis indicated that group C versus group A and group C versus group B were significantly different, respectively, in the incidence of HE (*χ*^2^ = 8.07, *P* = 0.004; *χ*^2^ = 9.44, *P* = 0.002) (Figure 4(b)), showing that the HE rate of group C was significantly lower than that in the other two groups. 47 patients developed mild HE, of whom symptoms were relieved after medical treatment. Seven patients developed severe HE, of whom 2 died of it.

Ten variables were evaluated as potential risk factors for HE in univariate analyses (Table 4). The multivariate analysis revealed that age (*P* = 0.001), previous HE (*P* = 0.014), Child-Pugh score (*P* = 0.002), reduction ratio of PSG (*P* = 0.015), and puncture site of the portal vein (*P* < 0.001) were independent predictors of HE (Table 5).

### 3.5. Postoperative Survival

The total mortality rate was 19.3% (33/171, group A: 18.2%; group B: 18.8%; and group C: 22.9%) (Table 2). The life table analysis showed that the 1~5-year survival rates were 82%, 78%, 78%, 75%, and 75%, respectively. The overall rate of survival among the three groups was not significantly different (*χ*^2^ = 0.364, *P* = 0.834) (Figure 4(c)). Among the 33 deaths, etiology included terminal hepatopathy in 21, uncontrolled esophageal gastric variceal hemorrhage in10, and severe HE in 2. Besides, 3 patients died of cardiac-cerebral vascular disease, which were not included above.

Nine variables were identified as potential prognostic factors of survival in the univariate analyses (Table 4). A multivariate analysis showed that previous ascites (*P* = 0.002), platelet count (*P* = 0.020), INR (*P* < 0.001), Child-Pugh score (*P* = 0.001), MELD score (*P* = 0.008), and puncture site of the portal vein (*P* = 0.003) were independent predictors (Table 5).

### 3.6. Liver Function

Changes in the Child-Pugh score among the three groups during pre-TIPS follow-up have been shown on Table 2. In group A and group B, the Child-Pugh score significantly increased during long-term follow-up (*P* < 0.05), compared with the pre-TIPS level. However, in group C, the Child-Pugh score significantly increased just in early times (*P* < 0.05) but has no significant difference during long-term follow-up after TIPS (*P* > 0.05). Additionally, there was significant difference among the three groups on the Child-Pugh score during 1-2 years and 3-4 years after TIPS (*F* = 1.922, *P* = 0.035; *F* = 2037, *P* = 0.020), and the Child-Pugh score in group C was lower than the other two groups.

## 4. Discussion

Two previous studies had reported the effect on the shunting branch of the portal vein after the creation of a TIPS [11, 13]. However, compared with the two studies, there were several significant differences in our study. First, as the previous studies reported, the portosystemic shunts were all created by the bare stent (BARD, Luminexx). The PTFE-covered stent (BARD, Fluency), by contrast, was implanted to create the portosystemic shunt in our study, while the bare stent (E-Luminexx; Bard) was also used to correct the angulation, bent, and covered from both ends of the covered stent if necessary. The primary patency rate of ePTFE-covered stent was higher than that of the bare stent, which had been proven in previous studies [14, 15]. Second, Bai et al. [13] reported that a 10 mm stent had been used for TIPS creation before October 2006 and that an 8 mm stent was used thereafter to avoid excessive portosystemic shunting. Chen et al. [11] reported that the stent was correctly implanted by initially dilating to 8 mm in diameter and then expanding to 10 mm if the PSG ≧ 12 mmHg. In our study, in comparison, we conventionally used an 8 mm stent to create a portosystemic shunt. An additional balloon dilation was performed if the PSG was ≧12 mmHg or the reduction in the PSG was <25%. A total of 15 6 mm stents was used in 12 patients with massive variceal hemorrhage and a Child-Pugh score of C10–12 to reduce the risk of hepatic encephalopathy. Third, patients were divided into two groups in the two studies, including the left branch and right branch. Instead, our study divided the patients into three groups, including the left branch, right branch, and intrahepatic bifurcation.

The majority of previous reports on TIPS were inclined to choose the right branch or the intrahepatic bifurcation to create the shunts [16, 17]. Some tended to agree that the rates of post-TIPS rebleeding and shunt stenosis among the three groups were not significantly different and that there was no necessity to choose the puncture site deliberately if the compliance of shunts was satisfactory [18]. Nevertheless, some achieved the conclusion that patients with the optimal stent position could acquire better long-term clinical efficacy [13]. Furthermore, some considered that the rate of shunt patency would be improved on a condition that the left branch of the portal vein was punctured [11, 19]. In our study, the results showed that the long-term rate of shunt patency on the left branch was significantly higher than that in the other two groups, which could be explained in detail by the following reasons. First, from an anatomical perspective, the left branch is the extending section of the trunk of the portal vein, and the trajectory between the middle hepatic vein and the left portal vein is straighter than that between the right hepatic vein and the right portal vein. Therefore, the plasticity of stent shunts would be more satisfactory because of less probability to develop the pseudointima hyperplasia and stent shunt stenosis. On the other hand, however, angulation or block would possibly appear at both ends of the stent, where high-velocity blood flow might cause the eddy, leading to excessive repair of the injured blood vessel endothelium and thrombosis [17]. Second, from a hemodynamic point of view, the laminar shear stress in the left branch of the portal vein induced less turbulence in a stent shunt, reducing the risk of thrombosis [20]. Third, from a physiopathological reason, the distance between the left branch of the portal vein and that of the hepatic vein is shorter, which facilitated the shunt creation and decreased the contact area between the stent and liver parenchyma, reducing the probability of pseudointima hyperplasia and growth of liver parenchyma into the stent [21]. Moreover, it might lead to thrombosis and stenosis in a draining veins, due to the longer stent shunt, the local turbulence caused by significant increase of pressure and blood flow velocity in the hepatic vein, and the intimal hyperplasia caused by chronic mechanical stimulation from stent.

When an ePTFE-covered stent graft is used, hepatic encephalopathy is a major complication associated with TIPS placement, which is the main concern for clinicians who choose TIPS to reduce portal hypertension. There was a high incidence of HE ranging 18%~45% after TIPS, along with some patients in a subclinical state [22]. It was reported that the incidence of HE could be decreased significantly if the left branch of the portal vein was punctured [11, 13]. Similarly, results in our study showed that the incidence of HE in the group of the left branch was significantly lower than those of the other two groups. As the two major venous circuits of the main portal vein, the bloodstream in the superior mesenteric vein and splenic vein has not been mixed adequately in the main portal vein before it is shunted into the right or the left branch of the portal vein, respectively, which means the blood in the right and left branch of the portal vein mostly comes from the superior mesenteric vein and splenic vein, respectively [23]. The blood in the superior mesenteric vein contains massive toxin compared to that in the splenic vein, especially ammonia, which means that the concentration of plasma ammonia in the right branch of the portal vein is higher than that in the left branch. It was reported that the concentration of plasma ammonia in veins were ranked as follows: superior mesenteric vein > main portal vein > splenic vein > peripheral vein, of which the significant difference had been proven [23]. However, these kinds of data from human patients are currently not available. From a physiopathological reason, the shunt of the left branch might decrease the loss of biological factors in the liver, which are rich in the superior mesenteric vein and play a crucial role in maintenance of liver function. Additionally, the shunt of the left branch could preserve most of the hepatic blood perfusion from the portal vein, which might alleviate the liver damage caused by shunting and reduce the risk of HE. In conclusion, choosing the left branch to create a shunt might reduce the risk of postoperative HE because of the lower concentration of plasma ammonia in systemic circulation.

TIPS is an effective method in controlling acute bleeding from varices, but without improving liver function. Liver failure, instead of the complications of portal hypertension, has become the primary cause of death for patients with end-stage cirrhosis after TIPS. Actually, we have also observed that most patients suffered from liver dysfunctions after TIPS. Therefore, we should pay more attention to improving the patients' liver function after TIPS. It had been reported that the liver function might be maintained by the establishment of a shunt on the left branch of the portal vein [11, 12]. Based on our results, the Child-Pugh score significantly increased just in early times (within 6 months after TIPS) compared with pre-TIPS but has no significant difference during long-term follow-up (3-4 years after TIPS) compared with pre-TIPS in group C (Table 2). Additionally, there was a significant difference among the three groups on the Child-Pugh score during 1-2 years and 3-4 years after TIPS, and the Child-Pugh score in group C was lower than those in the other two groups. This finding might be related to the fewer changes to blood perfusion caused by the shunt on the left branch of the portal vein, which maintains a better balance between the reduction of portal hypertension and the retention of hepatic blood perfusion from the portal vein. The left hepatic lobe, supplied for the blood by the left branch of the portal vein, accounts for 20%~25% volume of the whole liver. Therefore, compared with other puncture sites, impairment of liver function was minimized if the left branch of the portal vein was punctured. Moreover, combined with the biological factors mentioned above, liver function was maintained at the most extent by the left branch shunt. Thus, the post-TIPS life quality and long-term efficacy were improved effectively. Nevertheless, the result in our study did not show any statistical effect of the puncture sites on survival, possibly due to the small sample size and limited follow-up time.

Although the left branch shunt might lead to better clinical efficacy, there were some possible technical difficulties for the puncture of the left branch of the portal vein. When performing the left branch puncture, the metal guidance cube of RUPS-100 was usually bent more than 45°. In some cases, almost 90° heavy manual bending was required. The RUPS-100 was then turned 60° anticlockwise so that the puncture needle could get to the left branch of the portal vein from the hepatic vein, which was difficult and could lead to pain for the patients. In some cases, the left branch of the portal vein was too thin to be easily punctured.

The limitations in our study can be listed as follows. Firstly, the Viatorr stent, an ePTFE-covered device specially designed for TIPS, was not used in this study because it is not available in China until October 2015. However, the primary shunt patency rates in this study are also similar to those of TIPS created with Viatorr stents [9, 24]. Secondly, this is a retrospective study from a single centre over a short-time period and the baseline data of patients are limited. Thirdly, the choice of the hepatic vein has not been taken into account as the influence factor. Finally, there are still many other influence factors on post-TIPS efficacy.

In conclusion, choosing the left branch of the portal vein as the puncture site to create the shunt in TIPS with ePTFE-covered stents may improve the long-term shunt patency rate and decrease the incidence of hepatic encephalopathy effectively. Further studies are needed to determine whether the postoperative liver function and life quality would be influenced by the puncture sites of the portal vein.

## Figures and Tables

**Table 1:** Baseline characteristics of patients included in the study.

Variables	Group A (*n* = 88)	Group B (*n* = 48)	Group C (*n* = 35)	*χ * ^2^/*F*	*P* value
Gender (M/F)	67/21	33/15	25/10	0.924^∗^	0.630
Age (y)	52.6 ± 13.5	51.8 ± 12.5	50.2 ± 12.7	0.262	0.834
Etiology of cirrhosis				2.113^∗^	0.909
HBV	68	35	26		
HCV	5	2	3		
Alcoholic	8	6	5		
Idiopathic	6	5	2		
Main preoperative symptoms				0.615^∗^	0.735
UGI bleeding	69	40	29		
Refractory ascites	19	8	6		
Previous ascites	39	21	18	0.604^∗^	0.739
Previous HE	5	4	2	0.382^∗^	0.826
Hydrothorax	18	11	9	0.419^∗^	0.811
Platelet count (×10^9^/L)	80.22 ± 41.56	82.13 ± 34.82	78.65 ± 29.31	0.468	0.642
Haemoglobin (g/L)	81.32 ± 21.53	79.22 ± 22.96	78.65 ± 19.12	0.801	0.357
Serum albumin (g/L)	31.05 ± 6.23	32.54 ± 5.88	31.22 ± 6.07	0.710	0.429
Serum bilirubin (mmol/L)	23.21 ± 19.64	26.89 ± 18.76	25.63 ± 21.23	0.265	0.782
Serum creatinine (mmol/L)	78.75 ± 35.88	76.18 ± 30.21	77.57 ± 36.54	0.391	0.631
Prothrombin time (s)	15.18 ± 2.11	15.25 ± 2.15	15.22 ± 2.18	0.354	0.733
INR	1.41 ± 0.22	1.36 ± 0.34	1.39 ± 0.36	0.563	0.465
Child-Pugh score	7.46 ± 2.11	7.64 ± 1.89	7.42 ± 1.74	0.964	0.183
Child-Pugh class (A/B/C)	32/51/5	18/27/3	15/18/2	0.487^∗^	0.975
MELD score	11.81 ± 3.45	12.14 ± 4.01	11.92 ± 3.95	0.524	0.552
Pre-PSG	25.03 ± 4.42	24.40 ± 3.97	25.55 ± 4.12	1.104	0.343
Post-PSG	10.81 ± 3.10	12.14 ± 3.36	11.45 ± 3.22	0.672	0.525
Stent diameter (6/8 mm)	6/82	3/45	3/32	0.172^∗^	0.918
Lost to follow-up	9	6	4	0.165^∗^	0.921

HBV: hepatitis B virus; HCV: hepatitis C virus; HE: hepatic encephalopathy; INR: international normalized ratio; MELD: model for end-stage liver diseases; PSG: portosystemic gradient; UGI: upper gastrointestinal. ^∗^*χ*^2^ test

**Table 2:** Effects of the procedures on the outcomes.

Parameter	Group A	Group B	Group C	*χ * ^2^/*F*	*P* value
Total raw data					
Shunt stenosis	38.64% (34/88)	29.17% (14/48)	8.57% (3/35)	13.40^∗^	0.001
HE	35.23% (31/88)	39.58% (19/48)	11.43% (3/35)	10.20^∗^	0.006
Mortality	18.18% (16/88)	18.75% (9/48)	22.86% (8/35)	0.36^∗^	0.834
Rate of shunt patency (%)					
6 months	96.5	95.6	96.6		
1 year	85.6	85.7	92.7		
2 years	66.2	80.1	88.1		
3 years	44.9	72.0	88.1		
Rate of HE (%)					
6 months	13.0	18.2	2.9		
1 year	21.6	25.0	6.5		
2 years	37.1	34.1	6.5		
3 years	43.4	39.1	6.5		
Rate of survival (%)					
6 months	92.0	95.7	88.2		
1 year	88.4	91.3	85.1		
2 years	81.4	80.4	75.8		
3 years	78.6	74.7	75.8		
Child-Pugh score					
Pre-TIPS	7.46 ± 2.11	7.64 ± 1.89	7.42 ± 1.74	0.964	0.183
2–4 weeks	7.70 ± 1.39^a^	7.84 ± 0.83	7.79 ± 1.96^a^	0.991	0.084
3–6 months	7.63 ± 1.19	8.32 ± 1.33^a^	7.83 ± 1.62^a^	0.811	0.236
1-2 years	7.86 ± 1.65^a^	8.07 ± 1.94^a^	7.58 ± 2.03	1.922	0.035
3-4 years	7.91 ± 1.98^a^	8.11 ± 1.73^a^	7.39 ± 1.35	2.037	0.020

HE: hepatic encephalopathy; TIPS: transjugular intrahepatic portosystemic shunt. ^∗^*χ*^2^ test. ^a^*P* < 0.05 compared with the pre-TIPS level.

**Table 3:** Patterns of TIPS stent stenosis.

Pattern	Group A (*n* = 25)	Group B (*n* = 10)	Group C (*n* = 3)
Thrombotic occlusion	10	5	2
Intimal hyperplasia	7	2	1
Hepatic and portal venous end shunt stenosis	3	1	0
Abnormal angulation	5	2	0

**Table 4:** The results of the univariate analyses.

Variables	Shunt patency			HE			Survival		
	HR	95% CI	*P* value	HR	95% CI	*P* value	HR	95% CI	*P* value
Gender (M/F)	1.59	1.10–2.37	0.032	1.02	0.82–1.41	NS	1.31	0.82–2.05	NS
Age (y)	0.89	0.95–1.02	NS	1.04	1.02–1.06	<0.001	1.02	0.98–1.05	NS
Main preoperative symptoms (bleeding/ascites)	0.72	0.48–1.12	NS	1.27	0.78–2.04	NS	1.65	1.12–2.54	0.039
Previous ascites (yes/no)	0.89	0.65–1.43	NS	1.65	1.14–2.41	0.021	2.42	1.58–3.76	0.002
Previous HE (yes/no)	0.95	0.71–1.54	NS	1.74	1.22–2.60	0.016	0.71	0.46–1.12	NS
Platelet count (×10^9^/L)	1.01	0.98–1.03	NS	1.01	0.99–1.02	NS	0.99	0.99–1.0	0.025
Serum albumin (g/L)	0.96	0.94–1.02	NS	0.97	0.94–0.99	0.021	0.95	0.91–0.98	0.032
Serum bilirubin (mmol/L)	1.00	0.98–1.01	NS	1.00	0.99–1.01	NS	1.01	1.00-1.02	0.012
INR	0.84	0.51–1.43	NS	2.19	1.20–3.74	0.010	3.02	1.76–5.09	<0.001
Child-Pugh score	1.05	0.91–1.15	NS	1.37	1.26–1.49	0.006	1.49	1.35–1.65	<0.001
MELD score	0.97	0.90–1.03	NS	1.28	1.17–1.41	0.021	1.09	1.04–1.14	0.005
Reduction ratio of PSG (%)	0.99	0.98–1.01	NS	1.04	1.02–1.06	0.007	1.01	0.99–1.02	NS
Stent diameter (6/8 mm)	2.04	1.52–3.15	<0.001	1.97	1.56–2.58	< 0.001	0.80	0.54–1.20	NS
Puncture site of portal vein (bifurcation/right/left)	1.84	1.54–2.21	0.012	2.30	1.62–3.28	<0.001	1.75	1.32–2.42	0.004

HE: hepatic encephalopathy; INR: international normalized ratio; MELD: model for end-stage liver diseases; NS: nonsignificant; PSG: portosystemic pressure gradient.

**Table 5:** Risk factors from the multivariate analyses.

Variables	HR	95% CI	*P* value
Shunt patency			
Stent diameter (6/8mm)	1.92	1.41–3.04	0.007
Puncture site of portal vein (bifurcation/right/left)	1.78	1.48–2.20	0.015
HE			
Age (y)	1.05	1.02–1.07	0.001
Previous HE (yes/no)	1.54	1.12–2.46	0.014
Child-Pugh score	1.40	1.31–1.52	0.002
Reduction ratio of PSG (%)	1.08	1.06–1.11	0.015
Puncture site of portal vein (bifurcation/right/left)	2.26	1.60–3.25	<0.001
Survival			
Previous ascites (yes/no)	2.51	1.68–3.84	0.002
Platelet count (×10^9^/L)	1.06	1.04–1.09	0.020
INR	2.85	1.60–4.85	<0.001
Child-Pugh score	1.60	1.45–1.75	0.001
MELD score	1.10	1.05–1.16	0.008
Puncture site of portal vein (bifurcation/right/left)	1.94	1.52–2.64	0.003

HE: hepatic encephalopathy; INR: international normalized ratio; MELD: model for end-stage liver diseases; PSG: portosystemic pressure gradient.

## Data Availability

The data used to support the findings of this study are included in the article.

## Abbreviations

BNP: Brain natriuretic peptide
ePTFE: Expanded polytetrafluoroethylene
HBV: Hepatitis B virus
HCV: Hepatitis C virus
HE: Hepatic encephalopathy
INR: International normalized ratio
MELD: Model for end-stage liver diseases
NS: Nonsignificant
NYHA: New York Heart Association
PSG: Portosystemic gradient
TIPS: Transjugular intrahepatic portosystemic shunt
UGI: Upper gastrointestinal.
